# The Science of Nutrition

**Published:** 1897-06

**Authors:** 


### The Science of Nutrition.

That we are in error in our food economy is no longer a vague theme to be scoffed at by the thoughtless, noticed lightly by the reader, and believed in only by the scientist. It is a substantial fact, easily proved to those who would be doubtful. Professor Atwater, under the direction of the United States Agricultural Department, has given to the people such data in bulletins showing the result of his investigations that any one of ordinary intelligence may fully comprehend, so concise and complete are they.

While there is yet much to learn through experimentation, there is at hand sufficient information to enable us to make very important deductions for personal use. We are told by the best authority that we must come to the realization that "not merely our health, our strength, and our incomes, but our higher intellectual life, and even our morals depend upon the care which we take of our bodies, and among the things essential to health and wealth, to right thinking and right living, one, and that not the least important is our diet." It is our good fortune to have learned this—to be told that the science of food reaches to every point on the line of battle for existence.—*M. V. Shaler.*

